# A Practical Treatise on the Diseases of Children

**Published:** 1846-12

**Authors:** 


### PART II.—REVIEW.

### ARTICLE VI.

*A Practical Treatise on the Diseases of Children.* By James Milman Coley, M. D., member of the Royal College of Physicians, London, &c. &c. &c. Philadelphia: Ed. Barrington & Geo. Haswell. 1846. pp. 414. 8 vo. (From the Publishers).

The diseases of children have lately received a large share of attention from some excellent observers in the profession, and are just beginning to occupy that rank in the studies of practitioners, to which their frequency and severity, justly entitle them. Still we frequently find physicians in extensive and successful practice, who decline, in most instances, to prescribe for young children, turning them over to the care of nurses or old women, as more competent to take charge of their diseases. Not only is this the case with regard to their internal or *medical* diseases, but also in cases of surgical diseases as congenital hernia, nævi, &c., many of which we have seen neglected for months and years by the advice of medical men. Not only are physicians who neglect the diseases of infants in error, in supposing them more obscure and less amenable to treatment than those of adults, but they thereby, are led to neglect that part of their appropriate duties in which they might be most useful, for every one familiar with the diseases of infants, will agree that they are more easily recognized and more successfully treated than those of adults. Indeed if we except two points, viz: distinguishing cerebral from gastric and intestinal affections and detecting the existence of lobular pneumonia, we know of none of these affections requiring unusual skill for their diagnosis or treatment. The work before us is one of considerable pretension, the author—

"Having received a surgical as well as a medical education, and having been extensively engaged with operative surgery in the country, many years previous to my connection with the College of Physicians, and my residence in London, I have enjoyed singular opportunities of observing the origin and progress of surgical as well as medical cases, and acquiring that discrimination and manual dexterity, which are necessary qualifications in any one who undertakes to instruct others on subjects requiring a practical knowledge of both branches of the profession."—[Introduction.]

There is, however, one department which is entirely omitted viz: the hygenic treatment of children, for which he refers to the works of Drs. Underwood, Maunsell, and Evanson, and to which we would add Dewees, whose remarks on this head are extremely judicious.

The first division of the work is devoted to the surgical diseases of children, embracing every form of Hernia, Club foot and deformities, diseases of the eyes and eyelids, hydrocele, &c. The remarks upon each of these are brief, just, and present little new or remarkable.

We extract his discription of sanguine tumors of the scalp and refer such of our readers as may be desirous of further information on the subject to a monograph by Dr. Geddings in the Am. Jour. of the Med. Sciences.

"*Hydrocephalus Externus, or Cephalœmatoma.*—This is a tumour found on the head of the infant generally after a tedious labour, especially when the action of the uterus has been long resisted by the bones of the pelvis. The swelling consists of blood extravasated either between the integuments and the pericranium, or between the latter and the skull. The swelling varies in size from that of a hen's egg to that of a large orange.

"*Treatment.*—As this disease always disappears spontaneously by the gradual absorption of the blood, all we shall be required to do is to promote that process. With this view the following lotion or liniment should be prescribed:—

℞—Ammonia Hydrochloratis, ʒj.

Aquæ Distillatæ, ℥vj.

M. et fiant lotio.

℞—Potassæ Iodidi, ℈ij.

Adepis, ʒj.

M. fiant Linim. singulis noctibus tumori affricandum.

℞—Linimenti Hydrargyri, ℥j.

Fiat linimentum semel quotidie, parti affectæ illinendum.

"When the tumour is very large, Dr. Black advises that it should be punctured or incised to evacuate the effused blood.* Mr. Waffstaff, on the contrary, says this is a bad practice;† and I have never met with any case which required any other remedies than the external applications I have mentioned."

* "Edinb. Med. and Surg. Journal," 1841. † "Lancet," No. 743, p. 308.

Next in order follow diseases of the skin, and we lay before our readers the remarks on Vaccine, with the observation that the facts do not set at rest, as yet, the question in the sense supposed by Dr. Coley.

"When the vaccine vesicle has regularly passed through all these stages, the constitution is afterwards as securely protected against the attacks of small pox as if it had been affected by the natural or inoculated variola; and therefore the practice of repeating vaccine inoculation in the same individual is perfectly unnecessary, as was clearly proved by the observations of Dr. Jenner, and the accumulated experience of all intelligence vaccinators since his time. It has been proposed* that re-vaccination should be performed every five years, and if it should *succeed*, that it may be concluded that such repetition of the disease is not too frequent. The success of repeated vaccination is no proof that the constitution has lost its influence, as cow pox vesicles may be excited as frequently as the surgeon may wish, in most, if not all patients who ever had been susceptible of the disease; and the fact of two successive crops being generated, after the interval of a few days, by Mr. Bryce's test, is a proof that the system is not rendered insensible to re-vaccination by any particular interval of time. To admit that the prophylactic power of vaccination expires at a given time in any individual, and that it may be revived by re-vaccination, amounts to a confession that the primary operation had been imperfectly performed, or that the human constitution had undergone some degeneracy or modification since the discovery of Jenner. The occasional eruption of modified or natural or small-pox, even in the confluent form, after the regular vaccination, is only a proof of some peculiarity of constitution, which appears to prevail in certain families; but repeated vaccination, however perfect, will have no more effect in removing such idiosyncracy, than in altering the features of the patient.

"When vaccination is imperfect or irregular, it affords no certain protection. Its progress should therefore be carefully observed. One imperfection consists in the absence of the areola and secondary rose-rash; another in the formation of pus in the first instance instead of serum, accompanied with premature inflammation and decadence, and sometimes with ulceration. The cause of these irregularities is sometimes constitutional; as I have found them to occur repeatedly to the same individuals when vaccinated with healthy lymph, taken from different patients, which has produced the perfect disease in others. One of the most frequent causes of failure

* "Wilson on Diseases of the Skin," p. 91.

or imperfection, consists in the practice of inserting vaccine fluid taken at too late a period of the eruption, when it is beginning to undergo the transition from pellucid to opaque lymph. Another cause has appeared to me to consist in a defect in the virus, produced by its modification in a patient labouring under marasmus, or some specific disease. Hence I always recommend the supply to be taken from the vesicle at the earliest possible period, and from the most robust and healthy subject. When the inflammation and scab are remarkably small and premature, the patient cannot be considered safe, and should always be re-vaccinated with lymph taken early, and carefully selected. The success of vaccination depends in some measure on the manner in which the operation is performed. I believe the most certain mode of introducing the lymph is in a fluid state, by means of the lancet, on the point of which it is taken. Another effectual mode is by the aid of a few fine gilt needles, which have been immersed in the virus; and when it is inconvenient or impossible to procure fluid lymph, it may be used in a dry state, either on the point of the lancet or the needle. Ivory points are generally employed for this purpose, but they are objectionable on account of their requiring the puncture of a lancet before their insertion. After the operation is completed, the arm should be left exposed about ten minutes, to afford time for the punctures to dry. Much nicety is required in this simple proceeding, to prevent too much oozing of blood, which is apt to wash away the lpmph after it has been inserted under the epidermis. The nurse should also be desired not to wash the arm until the vesicle has passed through all its stages."

In regard to its protective power, however, when properly employed there can be no doubt.

"As to the protective influence of vaccination, that has been sufficiently proved to be quite as great as that of inoculated small-pox: but it must be observed, that when variola occurs in the epidemic form, more cases of modified small-pox will succeed the pestilence than would appear under ordinary circumstances; and the recurrence of small-pox after variolus inoculation was formerly found as frequent as modified small-pox is now after vaccination."

The following remarks upon the treatment of nævi materni are also of interest.

*Treatment.*—One of the best remedies for varicose excresence or nævus, is creosote, which may be applied about once a week to the whole of the diseased surface by means of a feather, or camel-hair pencil. By this application twice used,

I succeeded in removing this disease, which occupied thirty square inches of the skin of the abdomen, and which had been only temporarily cured by an extensive eschar produced by hydrate of potash. Creosote has besides this decided advantage over the latter remedy, namely, that of destroying the disease without disfiguring the skin. When the marks appear distinct from each other, every one, however minute, must be touched with the escharotic. Nitric acid will often succeed. I have ascertained from long experience, that this disease is much less apt to return or appear round the circumference, after it has been removed by escharotics, than when it has been extirpated by the knife; and therefore I have of late years discontinued to advise such an operation. Many other remedies have been adopted with various success by different practitioners. The mode of applying nitric acid adopted by Sir Benjamin Brodie, is by using a glass pen dipped into the acid and drawn over the diseased parts, or by puncturing the principal vessels, and afterwards introducing a litte acid with the pen.* Nitric acid, as well as creosote, leaves no mark. Dr. Sigmund applies repeated compresses saturated with liquor plumbi diacetatis (or what he calls acetum plumbi),† with success."

The chapter upon diseases of the mouth is full and interesting. Passing over his remarks on aptha as containing nothing new we would direct the attention of our readers to the subject of "Muguet or Mucosity," which is not often recognized by practitioners as a distinct disease.

"It consists of a secretion of white, thick opaque laminæ, adhering to the free surface of the epidermis, or mucous membranes. It is most frequently observable on the tongue, cheeks and fauces, either in distinct spots or patches, or covering the entire upper surface of the tongue. When attentively observed, it will be found to be preceded by slight diarrhœa, or dysentery; and it is a common concomitant or sequence of those fevers which originate in gastro-enteritric inflammation. Being readily conveyed to the delicate skin covering the nipple, it is a frequent cause of those excoriations which affect that part during lactation; but this contagious property is denied by Bacon and Billard. These white, milk-like deposits are readily separated from the epidermis by gentle friction, and are speedily renewed by the inflamed surface."

"*Treatment.*—As muguet is seldom found unconnected with disease in some other part of the alimentary passage, on which its continuance is dependant, I must refer the reader to those

* "Medical Times," Dec. 19, 1841.

† "British and Foreign Medical Review," Jan., 1844, p. 239.

affections. At the same time, I may observe, that the most simple attack will seldom entirely subside without the assistance of internal medicine, as castor oil, rhubarb, or sulphate of magnesia. In some obstinate cases, it may be found necessary to administer chloride of mercury and jalap, according to the directions given for aphtha. The only local treatment required is the frequent abrasion of the diseased secretion, by means of a lotion composed of ten grains of alum to an ounce of water."

The treatment of gangrene of the mouth consists in touching the sloughing points with concentrated nitric or muriatic acid, if the slough be extensive this should first be freely divided so as to allow the acid to come in contact with the living parts. Tonics and fresh air with cleanliness are essential, and in this region, quinine is often useful, as the defect of constitution, upon which the disease depends, is produced by that unknown cause of intermittent and remittent diseases called "malaria." Gangrene of the mouth he separates from "Cancrum Oris, or Sloughing Phagedæna of the Mouth," which latter is very often the effect of mercurial inflammation, and followed by immobility of the jaws, &c. The following is his treatment of this latter affection.

"This disease is rapidly cured at its origin by a few applications of the nitrate of silver. In the more advanced stages, it will be necessary to apply a lotion composed of diluted hydrochloric acid in the proportion of half an ounce or an ounce to a pint of water; and, when the sloughing and ulceration are rapidly extending, the nitric or hydrochloric acid should be applied undiluted, in the manner directed for the treatment of gangrene in the mouth. When the gums are irritated by stumps of teeth, these should be extracted. With respect to internal remedies, few will be required, except such as are necessary to support the strength, in conjunction with nourishing diet and occasional opiates and aperients. In many of the most unfavourable cases, Mr. Wallace, of Dublin, has effected a cure by the internal exhibition of sesquicarbonate of ammonia, in doses varying from five to twenty grains; and Dr. Hunt has successfully treated the disease by giving from twenty to forty grains of chloride of potash, in the course of twelve hours."*

The article on Dentition is very full, and some of the views of the diseases attendant on this state are original, whether just or not we leave our readers to determine.

* "Med. Gazette," April 7, 1843, p. 76.

*Dentition.*—A knowledge of the process which nature adopts in the formation of the deciduous teeth, is a necessary part of a medical and surgical education; and the general attention which dentition receives during childhood, renders it imperative that I should not omit any information on so interesting a subject in the present treatise.

"In the fœtus, about the third month, the margin of each jaw is found to consist of a channel, enclosing a mass of follicles, destined to form the future teeth. At the fourth or fifth month these follicles become more obvious, being then composed generally of eight distinct, globular capsules, which may with care, be elevated by the anatomist from the maxillary bone, so as to display the artery and nerve, which penetrate and form a pedicle for each. Minute perpendicular eminences, the rudiments of the alveolar processes, now present themselves; and as the fœtus approaches the period of its birth, the transverse septa are distinguishable, and the sockets become more complete. As soon as the separate cells are formed, the capsules no longer retaining their globular shape, accommodate their figure to their respective alveoli. Each jaw generally contains at birth ten of these partitions,—four for the incisors, two for the canine, and four for the molar teeth. Each capsule being now minutely examined, is discovered to consist of two membranes, the inner one being designed to construct the tooth and deposite its enamel, and the outer one to erect the alveolar processes; and as the operations of these membranes proceed, they are perceived ultimately to constitute the periostea for the respective teeth and sockets. Between these two membranes a fluid is observable, which is more or less considerable in proportion to the age of the fœtus;* and which, as the formation advances, is gradually absorbed. In the inner, or proper, capsule, about the fifth month the gums of the incisive teeth are perceptible at the upper part, next those of the canine, and afterwards those of the first molar teeth. To these primitive gums succeed solitary particles of bony matter in the capsules of the incisors and canines, and a congeries of osseous scales in the first molar membranes. The ossific process then proceeds from the crown of each tooth downwards, and the enamel is afterwards deposited by the inner surface of the same membrane, which had secreted the osseous matter. Should any constitutional disease occur during this delicate animo-chemical process of crystallization, it is rendered incomplete, and the teeth, which are ready for the reception of enamel at the period destined for its deposit, are either partially supplied with or totally deprived of it. Hence, in delicate children we find, after severe illness, the first incisors frequently present horizontal

* Mekel. "Manuel d'Anat. Generale Descript. et Pathol."

patches of imperfect enamel, alternating with bony furrows, entirely deprived of this covering.* The destined period for the deposition of the enamel having expired, this function of the inner membrane ceases; and as the crown of the tooth advances, the capsule, by which it was constructed, being no longer required, is gradually absorbed.

"The growth of the fangs now proceeds downwards, while the alveoli are simultaneously increased in depth; and when the teeth are explored during this process, the crown, or enamelled portion, is found complete externally, while the root remains towards the lower part still in a soft, pulpy, rudimentary state. The process of ossification commences at first externally, and when the outer portion of the crown is complete with its bone and enamel, the cavity is afterwards filled up; and as the root is being prolonged and ossified by the pulp, an inner, vascular membrane is observable, which constructs and lines the internal bony canals, through which the arteries and nerves proceed to their separate teeth. Thus we find that each complete tooth has, at the part which enters the alveolar process, both an internal and external membrane.†

"About the seventh month after birth the two middle incisors present themselves through the gums on the lower jaw, then the two outer incisors above and below, afterwards the eight molars, and, lastly, the four canines; making altogether twenty temporary or deciduous teeth. Some deviation from this proceeding occasionally occurs; the two lower incisors are now and then cut before birth, in which case they soon become deciduous; those on the upper jaw sometimes appear first, and, in some rare instances, no teeth are observed until the expiration of almost two years, which is the period generally occupied for the appearance of the entire set. In one case the two middle incisors, which were first perceptible so late as fifteen months after birth, became quite loose, projected, and were ready to be cast off at the age of twenty-two months. In this instance, as in most others, when dentition is remarkably delayed, a dangerous and protracted disease in the alimentary canal occurred.

"Irregularities in the first set of teeth are rare in consequence of their small size. When they do occur, the deviation usually happens to the cuspidati, which are last projected, and may be diverted from their proper situation in different parts of the bony palate. A deficient number, or an entire absence of teeth, may take place. Borelle met with a woman who, at sixty years of age, had never had any teeth.‡

"About the seventh year after birth, the second or permanent set of teeth begin to make their appearance. Previously

* See "A Practical Treatise on the Remittent Fever of Infants," by J. M. Coley, p. 156.

† "Billard." ‡ "Ibid."

to this event the incisors are found loose, being supported principally by the gums, their roots having undergone more or less absorption. When they remain firm at this period, the permanent incisors appear either in front or behind them, being always secreted in new and distinct capsules. The jaws having by this time acquired a great increase of size, the secondary teeth are found larger and more numerous. After the incisors are brought into view, amounting, as in infancy, to four in each jaw, eight bicuspides follow, and take the place of the primary molars; next eight molars shoot up some time from the tenth to the fourteenth year after birth; afterwards four cuspidati; and from the nineteenth to the twenty-ninth year, but usually at puberty, four wisdom teeth appear, one at each extremity of the jaws, and render the number of permanent teeth exactly thirty-two. These teeth, or four of the incisors or the canines, are sometimes wanting; and some instances are said to have occurred, where a third set of teeth has been cut at an advanced age.

"The gums of most of the permanent teeth are visible in the fœtus behind and below the milk-teeth.*

"I have in my possession a singular anatomical preparation, which I removed from the rectum of a woman about thirty years of age, who died of mortification of the omentum. It consists of a portion of the upper jaw of an extra-uterine fœtus, containing two incisor teeth and a cuspidatus, whose crowns are complete, and whose roots are enclosed in their respective capsules. Adjoining the cuspidatus is perceptible a primary molar tooth, which has been displaced and inverted, and continues adherent to the periosteum of the canine tooth by means of its investing membrane, which assumes the shape, and appears to have performed the office of a gubernaculum, about half of its fang having been absorbed. The particulars of this extraordinary case are recorded in the 'Edinburgh Medical and Surgical Journal,' Vol. vi. p. 50.

"*Diseases of Dentition.*—The following remarks of Guersant respecting the vulgar and unfortunate prejudices prevailing on the subject of dentition, and its supposed influence in producing diseases with which it has no connection, are so appropriate, that I am induced to introduce them for the notice of my readers:—

"On attribue dans le monde la pulpart des maladies de l'enfance au travail de la dentition. La difficulté d'observer les maladies du premier âge, et le peu de connaisances positives que nous avons sur cette partie de la pathologie, ont contribué, à enraciner cette opinion: et ce préjugé, resultant pe notre ignorance, est ensuite devenue populaire, commun, à tous les autres préjugés en medicine."†

* "Cyclopedia of Practical Medicine." Art. Dentition.
† "Dict. de Med." en 21 vol., t. 6. Art. Dentition, par M. Guersant.

"It is lamentable to notice the ignorance displayed by the profession, as well as the public, on this subject; every concomitant disease, the exact nature of which is not obvious to their apprehension, being attributed to the teeth. To enumerate all the complaints thus believed to be induced by dentition would be a waste of time.

"The diseases peculiar to dentition are such as arise from local inflammation. The rapid growth of the gums and alveolar processes, and the simultaneous activity of the dental capsules, require a corresponding supply of blood and nervous energy. Hence the flushings in the face, increased heat of the head, and cerebral excitement about that period. To this cause have been improperly ascribed those effusions of blood in the alveolar processes, which have been followed by destruction of the capsules and the alveoli. Two instances of this kind evidently occasioned by cold and accompanied by inflammation in the mucous coat of the alimentary canal, and a congested state of the liver, are related by Billard. In the first, an infant eighteen days old, was discovered an effusion of blackish fluid blood in three alveoli of the primary teeth. The incisive teeth and part of the germ, which was not ossified, floated free and detached in the effusion, which formed the tumour; the bony crowns of the teeth were softened, reddish, and almost macerated in the fluid. Some points of muguet were found at the inferior extremity of the œsophagus, red striæ traversed the surface of the stomach, and the mucous membrane at the end of the duodenum was thickened and tumefied. Near the valve of Bauhin six follicular plates, very red and much swollen, were met with; and the liver was gorged with blood.* In the second case, which also proved fatal to an infant twenty-six days old, the tongue and roof of the mouth were so affected by muguet that the nurse was unable to suckle him, and he vomited his food directly after he had taken it. Great heat of the skin and thirst occurred every evening, and the muguet extended itself. The gums in both jaws then became swollen, and deglutition almost impossible. The swelling in the upper lip made rapid progress, and the face was œdematous. Violent cough and a purple ecchymosis supervened before death. On dividing the swelling on the gums, it was found to be occasioned by blackish grumous blood, in the midst of which floated the dental gums, which, totally detached, escaped with the blood that flowed from the tumour. The stomach was contracted and wrinkled, and its mucous coat thickened and intensely red. The liver was distended with blood, and all the abdominal venous system in a very remarkable state of congestion. The tongue was the seat of a distinct œdematose swelling.†

* "Traité des Maladies des Enfans," p. 266.

† "Traité des Maladies des Enfans," p. 267, 268.

"These cases and dissections prove the co-existence of extensive disease in the stomach as well as the mouth, probably the result of exposure to cold at the latter end of autumn. As the attack commenced in each infant a few days after birth the process of dentition could have had no share in the cause of the disease. In short, effusion of blood into the alveoli, destruction of the processes, and exfoliation of the capsules of the teeth, cannot take place, except from external violence, or inflammation brought on by cold, or some constitutional derangement.

"When the milk-teeth are about to penetrate the gums, the absorbents remove the intervening substance, and thus, first one point of the apex of the tooth, and then another, is exposed to view. Sometimes the cuticle is so distended that inflammation supervenes, which, terminating in the effusion of serum or blood, detaches a portion of epidermis, which assumes the character of a small vesicle. Artificial aid is unnecessary, unless the symptoms of both local pain or constitutional irritation be present, when the inflamed or swollen gum should be divided with a crucial incision down to the presenting surface of the tooth.

"The principal diseases of the first set of teeth are caries and inflammation in the periosteal membranes, terminating in abscess, or what is commonly called *gum boil*. The first effect of inflammation in the periosteum is to excite pain, tenderness, and swelling in the gum adjacent to the tooth, and an effusion of fluid between the fang and its investing membrane, which is thus covered into a kind of cyst. Successive attacks of inflammation at length end in the formation of pus, which either bursts through the tumour in the gum, or may be artificially opened. In some cases after the abscess has burst or been opened, a fungus springs up from the diseased membrane lining the cavity. In other cases, the pressure of the abscess having produced absorption of a portion of the alveolar process at its lower part, it effuses its contents through the aperture thus formed, and the matter insinuates itself along the surface of the lower jaw, and forms an external tumour near its base. This tumour is at first hard and discoloured, but in the course of time it ultimately inflames, and bursting or being opened, leaves a puckering in the integument, which adhering to the bone, remains a permanent blemish. When the diseased tooth, which is the cause of the mishchief, is removed before external redness takes place, the tumour ultimately retires and leaves the skin unblemished.

"Children are subject to facial neuralgia from inflammation in the periosteal membranes of the teeth. This observes the same periodicity as it does in adults. What is called caries is a decay in the osseous part of the tooth, the nature of which has never been satisfactorily explained. It sometimes commences externally, at other it begins under the enamel, which is afterwards broken off and exposes the cavity. By those who believe that the teeth retain some minute and invisible kind of vascularity after they are completely formed, which others deny, caries is supposed to be a species of ulceration in the bone; yet no exfoliation ever takes place, and nothing like granulation has ever been observed in the carious cavity. When human teeth which have been long extracted from dead bodies, and when those formerly made with the tusk of the hippopotamus, have been artificially fixed in the human mouth and exposed to its secretions, they are found to undergo the same decay and present the same carious cavities as those found naturally formed in the respective sockets. Hence we may presume that these carious cavities are the result of some chemical process; and this supposition is strengthened by the fact that the process of decay is suspended by excluding the saliva and the external air by the introduction of pure gold, which is insoluble by the salivary secretion.

"When the digestive organs are so deranged that the supply of chyle is interrupted, and a species of scurvy is the result, the gums are apt to bleed from the slightest touch; and ulcerations also occur under such circumstances in the gums, where they are connected with the teeth.

"During the primary dentition it is not uncommon for the bowels to become constipated. This proceeds from the determination of blood towards the alveolar processes, and the consequent enervation of the alimentary canal. It is a most unfortunate mistake, into which medical men as well as the public are apt to fall to attribute a relaxed state of the bowels to dentition. It is impossible upon any sound pathological theory, to attribute either dysentery or diarrhœa to inflamed gums or alveoli; and in practice this gratuitous theory is followed often by the most unhappy and fatal results, particularly when the popular and routine custom of administering opium with astringents is adopted, or reliance is placed on needless lancing of the gums, and the muco-intestinal inflammation is neglected and unrelieved by appropriate remedies.

"*Treatment.*—Those inflammations of the gums, accompanied with muguet, which have been described by Billard, should be treated immediately by the application of a leech or two to the inflamed parts; and the lotion I have recommended, when speaking of muguet, should be regularly used. The congested state of the vessels of the stomach and liver will be most appropriately treated by small doses of castor oil, or rhubarb and magnesia. It must, however, be observed, that the only chance of rescuing the patient at so tender an age will be afforded the physician at the very commencement of so severe a disease.

"The inflammation in the periosteal membrane of the tooth is best treated by the application of a leech to the tumefied gum, which will generally subdue it at once, and prevent the suppurative process. The most safe and pleasant mode of introducing the leech into the mouth, is to pass a needle and thread tranversely through the animal, which should be afterwards placed within a glass tube. When suppuration takes place, the abscess may either be left to burst spontaneously, or it may be opened at a proper period with a lancet. As soon as the tenderness in the gum has subsided, the tooth should be extracted, as the patient cannot expect to enjoy any long immunity from a repetition of the abscess. Those cases which terminate in fungus, or in the formation of a tumour at the base of the jaw, should be treated in the same manner; as no other remedy than the extraction of the tooth can be relied upon to cure the fungus, or to prevent the disagreeable and lasting deformity, which an abscess connected with a diseased tooth would otherwise inflict on the face of the child.

"The only permanent cure for tooth-ache, occasioned by a decay in the tooth, is extraction. As the first set of teeth are only temporary, the process of introducing a round file, and removing the decayed surface, and filling up the cavity with gold, would be superfluous for a child. The front teeth ought to be extracted with a pair of small forceps, and the grinders by the key instrument, or by an instrument lately introduced by the dentists, which acts in the manner of a lever, with the assistance of a strong and practised hand. In using the forceps, the dentist should make use first of a slight rotatory motion, to separate the tooth from the alveolar process, and then extract it; and when the key instrument is employed, he should apply the left fore-finger on the middle of the convex surface of the claw of the instrument, to prevent its slipping while he is extracting the tooth. For this purpose, the claw will be found greatly improved by the addition of a small stud at the part above-mentioned; and with the key instrument, thus improved, the smallest stump may be removed with facility, provided the gum has been previously lanced to a sufficint depth, and with much more ease and despatch than by the barbarous instrument, called a punch, which ought long ago to have been exploded.

"The front permanent teeth are subject to irregularity, and require attention from seven to eighteen years of age. When the incisors are forced out of their proper situations by any of the temporary teeth, the latter should be removed; and when the cuspidati project, and endanger the upper lip at any time under eighteen years, the adjoining bicuspis should be extracted. After the patient is above eighteen, the irregular canine tooth should be removed, as there is no chance beyond that age of the errant cuspidatus occupying the place of a bicuspis the growth of the jaw being then complete.

"A fungous and bleeding condition of the gums connected with scurvy, is only to be cured by proper food and attention to the digestive organs. The food should consist of fresh meat and vegetables, and the child should take a mixture composed of diluted sulphuric acid and disulphate of quina, drinking lemonade through the day as a beverage. The bowels should be relaxed every third day with chloride of mercury and jalap. A severe case of this kind, with other alarming symptoms of scurvy, as vesications and ulcers in different parts of the body, occurred in my practice lately in a boy, who had been at a cheap school, where the diet consisted almost entirely of red herrings and other salt fish. The ulceration in the gums may be treated with a lotion composed of three or four grains of nitrate of silver to the ounce of water.

"The most proper remedy for facial neuralgia, before the first set of teeth have fallen out, when the teeth are decayed, is extraction. When the disease occurs afterwards, it should be treated as it is in adults, by means of disulphate of quina, which may be given in the dose of one grain three times a day, and, should that fail, by five minims of the solution of the arsenite of potash once in six hours. It must be observed, however, that when there exists any chronic inflammation or abscess in the periosteum of the tooth, the only effectual remedy is the entire removal of the tooth.

"Those cases in which constipation is present and obstinately continued by the process of dentition, must be treated as long as that condition prevails, by a tea-spoonful of castor oil, or a small dose of salts and senna, every morning. No fears need be entertained respecting the habit of giving repeated aperients, while dentition is proceeding, as the bowels assume their natural functions, as soon as the teeth are developed. When the costiveness is unusually obstinate, a dose of chloride of mercury and jalap must be given every third or fourth morning."

We would particularly recommend to the attention of practitioners the author's account of "Dyphtherite, or Membranous Sore Throat," of which several examples have been met with lately in this city.

"It begins with inflammation of the mucous membrane of the soft palate, tonsils, and pharynx, terminating in the secretion of a false membrane, *without any ulceration or destruction of the true skin.* As the inflammation advances, it is apt to extend to the larynx, and to produce the symptoms and fatal results of croup. In one form of the disease, gangrene or sloughing of the inflamed parts takes place, particularly in children of feeble constitution. The attack begins with a little fever, attended with a slight difficulty in swallowing. On inspecting the throat the tonsils are perceived to be swollen, and small portions of white or yellowish lymph may be seen, resembling muguet, on different parts of the soft palate and pharynx. After a short time these deposits of lymph assume a grey colour, and acquire an offensive odour; and a copious discharge of saliva flows from the corners of the mouth. At this period the cervical glands become inflamed and swollen. At length the grey lymph, constituting the false membrane, either falls off in a mass, and is ejected through the mouth, or it is separated in fragments and discharged by degrees, and is often reproduced."

"In the more malignant cases, the disease extends into the air-passages, producing symptoms of laryngeal and tracheal inflammation. First hoarseness is observed; then a harsh, suffocating cough, accompanied with a croupy sound and an anxious expression, followed by a pale, cadaverous countenance, with the eyes sunk in their sockets; hurried and feeble pulse, cold skin; and terminating, when unrelieved, in irresistible stupor, a purple colour of the lips, face, and extremities, and speedy death. When the bronchial tubes are visited by this disease, the cough becomes more frequent, the breathing more rapid, and accompanied with a mucous or rattling sound, and the patient sometimes expectorates shreds or tubular portions of lymph presenting a membranous appearance."

"*Treatment.*—As the danger of this disease is in proportion to the nature and extent of the false membrane, our principal reliance must be placed on local remedies. Of these the most effectual are hydrochloric and nitric acids, either of which may be conveyed to the diseased parts by means of sponge or linen rag fastened to a piece of cane or whalebone. The acid should be rubbed or pressed firmly on the surface of the parts affected, so as to insure its contact with the inflamed membrane and the detachment of the lymph. In very slight cases resembling muguet, a lotion composed of two grains of bichloride of mercury, or ten to twenty of nitrate of silver, to an ounce of distilled water, will be found sufficient to separate the excretion and remove the subjacent inflammation, the progress of which must be carefully watched and promptly arrested. The operation of these powerful stimuli on the congested and inflamed surface is that of producing contraction, and restoring the natural action of the minute vessels."

When all other means fail laryngotomy has sometimes been resorted to with success. A method of operating, quite novel, is recommended, said to have been invented by Mr. Hilton, and of which the description is extracted from Guy's Hospital reports. It is as follows:

"'In this operation I used a curved trochar and canula, the canula being oval from side to side, and the trochar lancet-shaped, much flattened above and below, and cutting at its point and edges. This instrument may be passed through the crico-thyroid membrane into the larynx, or through the trachea with the greatest facility, the larynx being held steady by the surgeon's left hand: indeed, it is scarcely necessary to divide the skin with a lancet before attempting its introduction; yet, with the circumstances permitting, I think that a good previous step. The forms of the cutting instrument and the canula are so adapted that the canula presses upon the whole of the cut surface, and thus prevents any internal bleeding; and, further, as regards, laryngotomy through the crico-thyroid membrane, the oval outline of the canula is the form best adapted to the form of the space between the cartilages. It is said some persons cannot bear a canula in the larynx or trachea. I apprehend that when this inconvenience arises, it occurs from the end of the canula touching the posterior part of the larynx or trachea, a point easily determined at the time by knowing the length of the canula, and passing a probe to its then internal extremity. This contact it is difficult to avoid with a straight or slightly curved canula: and such a one is also very liable to be blown out of the larynx by the patient coughing. With the intention of avoiding these inconveniences, it is better to use a trochar and canula very much curved, which, when introduced, hooks itself into the larynx or trachea, and is very secure in its position, with its internal aperture presenting itself completely to the centre, and in the axis of the trachea, in which it is placed, and so offering the greatest facility to the passage of the air during respiration and for the exit of mucus.

"'This operation with the trochar and canula may be done well, and almost in an instant, by any medical man,—is not in itself in any way dangerous,—not painful,—and almost invariably gives immediate relief, imposing very little inconvenience on the patient at the time or subsequently: and when the necessity for the artificial opening no longer exists, the aperture closes with facility, and leaves but little cicatrix.'"

It is obvious that this operation would only be admissible where no foreign substance or false membrane required to be extracted.

From the remarks on flatulence we extract the following prescription for that troublesome affection.

"℞—Magnesia, ʒss.

Sachari albi, ʒs.

Olei Carui, gtt. j.

Spiritûs Ammon fœtid. ʒss.

Tinct. Sennæ, ʒss.

Liquoris calcis q. s. ut fiant ℥jss.

"Half a tea-spoonful, or a tea-spoonful, to be taken when the flatulence is troublesome."

We will pass by the subjects of remittent fever, cholera, and the diseases of the stomach and bowels, since our object is rather to enrich our pages with valuable facts, than to point out imperfections in the work. It is quite obvious that the remittent fever, prevalent to a great extent in these western states, and common to children and adults, is entirely unknown to our author. The same is also true to nearly the same extent, in regard to cholera infantum, which is unknown in the middle and northern parts of Europe.

From the chapter on Nasal Catarrh we take the following which, "if true," may be useful to the horse as well as the physician.

"The indication we have to fulfil, is to restore tone to the mucous membrane, which may be affected by the following mixture:—

"℞—Cupri Sulphatis, gr. iv.
Quinæ Disulphatus, gr. vi.
Acidi Sulph., dil. m. iij.
Aquæ Distilatæ, ʒiij.
M.—Capiat cochl. i. minimum bis die.

"Should the discharge not be diminished within a few days, the dose of the sulphate of copper must be increased gradually until slight sickness is produced. The modus operandi of the copper, to which I attribute the principal benefit of this mixture, is that of restraining the mucous secretion from the follicles of the stomach. By continuous sympathy, this astringent operation of the medicine is extended to the Schneiderean membrane, which rapidly reduces its mucous and purulent discharges. This effect of sulphate of copper on the Schneiderean membrane of the horse, is still more rapid and remarkable; as any one may observe by exhibiting one drachm every day to that animal, afflicted with the most chronic inflammation and muco-purulent discharge from the nostrils, uncombined with glanders. He will find in the course of a week, the mucous membrane of the nose, lose its dark red, inflamed appearance, and resume its natural grey colour, which favorable change will be accompanied with a total cessation of the morbid discharge."

Omitting a great number of interesting articles we extract the following on "spasm of the glottis," or "thymic asthma," with the remark that the view here taken is the one now almost universally adopted.

"*Cerebral Croup, Laryngismus Stridulus, or Spasm of the Glottis.*—This disease was minutely described by Dr. John Clark, in his Commentaries on the Diseases of Children; and his description is so accurate, that I am induced to extract it verbatim:—

"'This convulsive affection occurs by paroxysms, with longer or shorter intervals between them, and of longer or shorter duration in different cases, and in the same case at different times.

"'It consists in a peculiar mode of respiration, which it is difficult accurately to describe.

"'The child having had no apparent warning, is suddenly seized with a spasmodic inspiration, consisting of distinct attempts to fill the chest, between each of which a squeaking noise is often made; the eyes stare, and the child is evidently in great distress; the face and the extremities, if the paroysm continue long, become purple, the head is thrown backwards, and the spine is often bent, as in opisthotonos: at length a strong respiration takes place, a fit of crying generally succeeds, and the child, evidently much exhausted, often falls asleep.

"'In one of these attacks a child sometimes, but not frequently, dies.

"'They usually occur many times in the course of the day and are often brought on by straining, by exercise, and by fretting, and sometimes they come on from no apparent cause.

"'They very commonly take place after a full meal, and they often occur immediately upon waking from sleep, though before the time of waking the child had been lying in a most tranquil state. As the breathing is affected by these paroxysms, the complaint is generally referred to the organs of respiration, and it has been sometimes called chronic croup, and is altogether of a convulsive character, arising from the same causes, and is relieved by the same remedies as other convulsive affections."

"'Accompanying these symptoms, a bending of the toes downwards, clenching of the fists, and the insertion of the thumbs into the palms of the hands, and bending the fingers upon them, is sometimes found, not only during the paroxysm but at other times.

"'Clenching the fist with the thumb inserted into the palm of the hand, often exists for a long time in children without being much observed, yet it is always to be considered as an unfavorable symptom, and frequently is a forerunner of convulsive disorder, being itself a spasmodic affection.'*

"This disease seldom appears before the third month, or after the third year. This may be accounted for partly by the gradual increase in the aperture of the glottis, which continues to proceed until puberty. It is a frequent consequence of dysentery, and concomitant with marasmus, especially in children who are dry nursed. Dr. Clarke, in his above description of the disease, has omitted to mention a swelling sometimes migratory and sometimes permanent, on the back of the hand or foot, and sometimes on the face, which commences rather suddenly after one of the fits in the advanced stage of the complaint. The swelling consists of serum, which is effused into the cellular membrane, and which appears to me to result from the temporary plethora and debility in the extreme vessels, occasioned by the obstructed circulation in the lungs during the paroxysm. This swelling is of the same nature as that which appears suddenly on the arms, the hands, the feet, and other parts of the body, and which migrates from one place to another, in patients advanced in life, and who have been reduced in strength, and been suffering a long time with repeated paroxysms of alarming obstruction in the pulmonary circulation, arising from fatal disease in the heart and pericardium.

"Various opinions have been formed respecting the nature and cause of this alarming disease. Dr. Clarke, and most other writers, have considered it as a spasm of the glottis. Dr. Hue Ley believed it to arise from paralysis of the recurrent nerve, occasioned by the pressure of enlarged cervical glands; and it is the opinion of Kopp, Hirsch, and most German authors, that it is due to the enlargement of the thymus gland. On the other hand, Caspari, Pagenstechr, Roesch, Hackman, and most British physicians, as well as Dr. Clark, have considered the disease as purely spasmodic. As the disease proceeds, it generally terminates in epileptic convulsions, especially in delicate infants; and it is not uncommon for the child to expire during one of these attacks. On this account Dr. Clarke supposed that in every case the brain is at the time organically affected either directly or indirectly. He believed this organ to be directly affected when the spasm arises from perenitis or hydrocephalus; and, indirectly, when it proceeds from an overloaded stomach indigestion, inflammation in the lungs or pericardium, from the pressure of glanular swellings, or when it occurs during the progress of infantile remittent fever or marasmus. In proof of this opinion, Dr. Clark states that he found in one patient after death, a collection of purulent matter in the pericardium, and in another fullness of the vessels and water in the ventricles of

* Commentaries on the Diseases of Children, by John Clarke, Esq., M.D., p. 86—89.

the brain. In addition to some cerebral affection, Kyll attributes the spasm of the glotts to an inflammation of the cervical portion of the medulla spinalis, and to an alteration in the structure of the cervical and thoracic glands, which compress the pneumogastric nerves; and, according to Dr. Marshall Hall, it may originate in inflammation of the gums, disease of the brain, or derangement in the alimentary canal. There is no doubt the cause of this disease is occasionally traceable to one of the nervous centres. That cases, however, will be found to originate in the last cause Dr. M. Hall has enumerated, we have abundant proof every day; and I hope to be able to show that it is almost invariably the cause of the disease. In a fatal case, which occurred in my own family, the only morbid appearance found on dissection was a large exostosis growing on the inner surface of the occiput, which compressed the cerebellum and produced chronic inflammation of the dura mater. In this patient no disease was discoverable either in the cervical or thoracic glands. In another fatal case, also in my own family, in which the spasm was almost continual before death, the only morbid appearance found on examination was inflammation in the left phrenic nerve, as it passed over the pericardium. With respect to dentition, I have never found the disease in any manner connected with that process. In one patient the gums had been lanced most unmercifully, down to the alveolar processes, by the practitioner in attendance, and salivation had been induced without any relief having been afforded; and as the case is full of interest, and tends to confirm the view I have long taken of the general cause of spasm of the glottis, I will presently relate the particulars of it. There is no doubt that certain infants are liable to this disease from various causes; but most of the cases, except the two fatal ones I have mentioned, which have occurred in my practice, have arisen from an excited state of the layrngeal nerves, produced by the pressure of undigested food in the stomach or duodenum, or some portion of the other small intestines. In these cases, when the stomach has been the original seat of the disease, the paroxysm has always occurred after a meal; when the duodenum or the other small intestines have been its seat, the fit has not taken place in less than an hour after taking food, when it has always been immediately followed by epilepsy. The morbid condition of the stomach has been preceded by imperfectly cured remittent fever, or by a neglected state of the bowels; and on examining the intestinal discharges, after the operation of opening medicine, they have been found undigested, no alteration having been made in the appearance of the food during its passage along the alimentary tube. Hence, when panada has been the food given to the child, the evacuations will be found to consist of the bread with which it was made, perfectly unaltered. In these cases, I apprehend there is a deficiency of gastric juice and mucus in the stomach, which is from that cause rendered irritable by the presence of food, for which it is not properly prepared; and when the crude aliment is propelled into the duodenum, and the natural secretions of that bowel, and the discharges from the liver and pancreas are either absent or defective, it appears that its mucous coat is impatient of its contents, and the nerves with which it is supplied excite the distant muscles of the larynx into spasm, on the same principle that the morbid secretions in cholera, irritating the mucous surface lower down the canal, excite the muscles of the legs and abdomen into spasmodic action; and the successful practice of Hackmann, in the administration of musk and oxide of zinc in chronic cases, appears to illustrate this analogy. As soon as the digested food has passed from a healthy stomach into the duodenum, the pancreas and the biliary ducts pour out their tributary streams, which unite with the chyme and the mucous secretion of that intestine effused from its numerous follicles. When the concurrence of these secretions is prevented by any cause, the symptoms which have occurred to my observation, when I had reason to suppose the food had arrived at the duodenum, have led me to believe that the inner surface of that intestine had in such cases acquired a morbid sensibility, which had deranged the healthy functions of the paria vaga and the nervous centres.

"As far back as the year 1723, Richa',* and 1726, Verduis,† described this disease, and referred its cause to hypertrophy of the thymus gland. In 1830, Kopp‡ published a memoir on this subject, and as he also considered the thymus gland to be in fault, the affection was called after him, "the thymic asthma of Kopp." Frank also asserted that anatomists often found the thymus and bronchial glands tumefied. In 1836, Dr. Hugh Lee, to whom I have before referred, published an essay on this disease, which he denominated laryngismus sidulus. He adopted the same view with respect to the pressure on the laryngeal nerves in every instance; and he endeavoured to account for the crowing inspiration, by supposing that the pressure of the enlarged cervical glands, &c., produced paralysis in the nerves in question. Dr. S. Merriman also appears to have entertained a belief that the disease is occasioned by the pressure of glandular swellings.|| On the contrary, Dr. Kerr§ denies that pressure on the nerves, or dentition, has any effect in producing the complaint; and Dr. Marshall Hall, one of the best physiologists of the day, thus expresses himself:—

* "Constitutiones Epidemicæ Taurinenses." † 'Dissertatio de Asthmate Pnerorum.'
‡ "Denkwardigkeiten in der Acteylichen Praxis."
|| "Underwood on the Diseases of Children," ninth edition, p. 142.
§ "Edinburgh Med. and Surg. Journal," vol. lviii., pp. 334 to 335.

"'It has been recently attempted to found the pathology of this interesting disease upon observations such as that adduced by Dr. Merriman, but I think unsuccessfully.

"'In the first place, as far as my memory and judgement serve me, the cases adduced to support this view are not cases in point, but, in reality, cases of other diseases.

"'Secondly, supposing pressure upon the par vagum to exist, it would induce totally different phenomena from those actually observed in this disease, and it would not explain the *series* of phenomena which actually occur in it; for,

"'1. Such pressure would induce simple *paralysis.*

"'This would, in the first place, affect the recurrent nerve and the dilator muscles of the larynx: it would induce a partial but *constant* closure of that orifice,—a permanent state of dyspnœa, such as occurred in the experiments of Legallois, or such as is observed to be excited in horses affected with the '*cornage*' or *roaring.*

"'Secondly, it would induce paralysis of the inferior portion of the pneumogastric nerve, with congestion in the lung or lungs, and the well known effects upon the stomach of the division of this nerve.

"'2. The disease in question, on the contrary, variously designated 'peculiar convulsion,' 'spasm of the glottis,' &c., &c., is obviously a *part* of a more general *spasmodic* affection, and frequently induced, most frequently comes on in the midst of the first *sleep*, in the most *sudden* manner, receding equally *suddenly*, to return, perhaps, as before, after various intervals of days, weeks, or even months. Very unlike paralysis, from *any* cause!

"'3. It not unfrequently involves, or accompanies, as I have said, *other* affections, indisputably *spasmodic*, as distortion of the face, strabismus, contraction of the thumbs to the palms of the hands; of the wrists, feet, toes; general convulsions! sudden dissolution! a series of phenomena totally unallied to paralysis.

"'4. Indeed, the larynx is sometimes absolutely *closed*, an effect which *paralysis* of the recurrent nerve, and of its dilator muscles, *cannot* effect.

"'5. Paralysis, from the pressure of diseased glands would be a far *less curable* disease, a far *less variable* disease, a far *less suddenly fatal* disease, than the croup-like convulsion.

"'Thirdly. Almost all recent cases are at once relieved by attention to three or four things, viz.:—the state, 1, of the *teeth;* 2, of the diet; 3, of the bowels; and 4, by change of *air.* They are as obviously produced or reproduced by the agency of errors in one or more of them.

"'Fourthly. In fact, the croup like convulsion is a *spasmodic* disease, excited by causes situated in the nervous centres, or eccentrically from them. In a case of spina bifida, a croupy and convulsive inspiration was produced by gentle pressure on the spinal tumour. In cases from teething, the attack has been produced and removed many times, by teething, and by freely *lancing* the teeth, by crudities, and by emetics and purgatives, by change of air, &c.

"'Fifthly. There is a series of facts which prove the connexion of this disease with other forms of convulsions in children, and with epilepsy in the adult subject.

"'Sixthly. In protracted cases, congestion and effusion within the head occur, as *effects* of this disease.

"'Lastly. Innumerable cases of undoubted croup-like convulsions have occurred, in which no enlarged glands would be detected in any part of the course of the pneumogastric nerve.'"

"*Treatment.*—When the patient is florid and plethoric, especially when general convulsions have followed the attack, and the head is hot, a few leeches must be applied to one of the temples, and a purging dose of chloride of mercury and jalap should be given every second or third day, according to the severity of the paroxysms. The proper proportion of the chloride will be one grain to four of jalap for an infant one year old, the dose being double that strength for the second, and treble for the third year. The evacuations must be examined, when they will be found to contain undigested food, and probably a quantity of highly offensive, dark coloured mucus. Although I have never seen any benefit from lancing the gums, yet should they be prominent and inflamed, from the pressure of the subjacent deciduous teeth, they may be lanced. It must, however, be borne in mind, that when the gums are tumefied and inflamed, the relief to be expected from their incision will be diminished in proportion as the child is advanced in age. In performing this operation, all that is required will be to divide the gums crucially down to the teeth, which should always be distinctly perceived by the touch to be near the surface previously to the operation. If the child is dry-nursed, a wet-nurse should be provided; or, if this cannot be done, the food should consist only of barley-water, or thin gruel prepared from grits, and passed through a fine sieve. When the child is three or four month old, a wine-glassful of this food will be sufficient about once in two hours during the day. No animal food, nor rusks, nor bread, nor milk, should be allowed such an infant, until all its primary incisive teeth have advanced through the gums, when, if the disease has subsided, he may take some of those articles of diet. He should be taken into the fresh air every fine day, and not confined to a hot apartment. His head must be kept cool, and if he has not sufficient hair to dispense with his cap, one as thin as possible should be worn. I have often known severe paroxysms brought on by a heated room, and by too much clothing about the head. Especial care must be taken to avoid mental pain or excitement, as the attacks of croupy inspiration are very apt to be induced by sudden passion.

"The opposite variety of the disease,—that which appears in delicate, pale, and sickly infants—never requires bleeding. The powder, composed of chloride of mercury [illegible] jalap, must be administered every second or third morning, and on the intermediate mornings a tea-spoonful of castor oil. This plan must be pursued until the stools become natural in smell and appearance: for as long as they consist of crude, undigested materials, or present the vicious, offensive mucous secretions peculiar to this disease, which I have described as resembling undiluted white lead paint, the fits of crowing inspiration will not fail, from time to time, to occur. So long, too, as these alvine discharges continue, emaciation will proceed, and the muscles will remain soft and flaccid. Such patients, when the state of the intestinal secretions and discharges are neglected, and appropriate treatment is not adopted, soon become afflicted with marasmus; the paroxysms increase in frequency and severity, and epilepsy following every attack, in a short time exhausts, and ultimately destroys life. These feeble and pallid infants receive no relief from lancing of the gums, nor, as I have said before, from other local bleedings; on the contrary each successive loss of blood increases the malady; and, when the seat of the disease and the proper treatment are overlooked, such infants become miserable objects, having one or both hands and feet hanging down, and distorted with a continued contraction of the flexor muscles; anxious, irritable, and appearing every moment to expect a fit, which each time threatens to destroy life. As soon as the morbid secretion of mucus has disappeared, the chloride of mercury and jalap must be discontinued, and the castor oil should be regularly repeated, either every morning, or every second morning, to obviate costiveness, and thereby prevent a recurrence of the attacks. At this period, great benefit will be derived from pure air; and, if the child should live in the town, he should be removed into a warm and dry situation in the country. The diet suitable for this variety will consist of barley-water or thin gruel, strained, till the first eight front teeth have escaped through the gums, when he may be allowed, with advantage, mutton or veal broth, and the yolk of an egg, boiled two minutes, once every day. In the feeble children subject to this disease the process of dentition begins prematurely; and this is one cause of the appearance of the symptoms. The determination or relatively increased flow of blood to the alveolar processes and dental capsules, necessary for the development of the teeth and the deposit of enamel, being, in these weak and irritable children, an exhausting effort of the vital principle, which deprives the alimentary canal of its due and accustomed share of nervous energy, the secretions in the chylopoietic viscera and the peristaltic action of the stomach and intestines are diminished, and indigestion and costiveness are the result. The vital processes of chylification and sanguinification are thus diminished in girls of feeble constitution, during the development of the ovaries preparatory to the process of menstruation. In these the excitement of the reproductive organs, necessary for their rapid growth and maturity at the destined period alluded to, attracts to those organs a temporary excess of the vital principle at the expense of the organs of supply; in consequence of which the natural action of the bowels is retarded, the various secretions necessary for nutrition are interrupted, the blood is ultimately deprived of its due portion of fibrine and red globules, and the condition of the sanguiferous circulation called anæmia, is established. The same principle will be found to operate in pathology, as well as in the natural development of particular organs. In all those varieties of mania, which are of a remittent character and in which a connection may be traced with the digestive organs, we find the bowels in so torpid a state, as to require the frequent or constant use of purgative medicines. So, also, in some, especially scrofulous inflammations, to which the eye is subject, we find, during the continuance of the local excitement, such a torpid state of the alimentary canal, as requires a constant administration of purgatives.

"In some scrofulous children, I apprehend a state of chronic inflammation in the muciparous glands and villous tunic of the small intestines, similar to what takes place in the mucous coat of the eye in purulent opthalmy, occurs, and is the source of the copious discharge of muco-purulent matter, which I have observed in some cases of disease, accompanied with spasm of the glottis. Such cases require the same persevering use of castor oil, or other purgative, as I have recommended; and, when curable, do not admit of cure or relief from any other treatment. The stimulus of purgative medicines, in such cases, carries off these morbid secretions, and, at the same time, excites the secretory vessels into more healthy action."

"*Hooping-Cough, or Pertussis.*—This is one of those diseases which on account of its frequent occurrence, is unfortunately too often left to the exclusive management of nurses. The consequence is, that many children annually perish from bronchial or pulmonary inflammation, excited by neglect or improper treatment, or remain sufferers through after-life from asthma, generated by dilitation of the bronchial ramifications, or by vesicular emphysema.

"Most English physicians consider hooping-cough to be contagious as well as epidemical. French medical writers believe it to be epidemical. It rarely attacks young infants and old persons, although no age is exempt from it. One of my patients, who was only three months old when the disease began, died from convulsions, excited by the cough, within fourteen days. Pertussis attacks the individual only once in his life, commencing in the autumnal, winter, or vernal months, and usually subsiding during summer. It has two principal stages, the Catarrhal, and the Convulsive, or Spasmodic; and some writers add to this the stage of decline. The first stage commences in some patients as a common catarrh, with a frequent, tickling cough, and slight fever; in others, with acute laryngitis, or croup. This stage continues from a week to a fortnight, and is succeeded rather abruptly by the second, which is denoted by the characteristic cough, whence the disease takes its name. This peculiar cough consists of successive, involuntary suffocating expulsions of air from the air-passages, succeeded by a long and sonorous inspiration. These paroxysms of convulsive coughing are often so violent, as to occasion epilepsy, or effusion of blood beneath the conjunctival membrane of the eye, or from the nose or ears. The patient, under these circumstances, becomes almost black in the face, and feels a sense of approaching suffocation, until inspiration returns. In severe cases, several fits of convulsive coughing occur in succession, until the child is quite exhausted, and almost senseless. In most cases, when the mucous membrane of the minute ramifications of the bronchi, or the pulmonary air-cells are the seat of the specific inflammation, the stomach is acted upon mechanically by the convulsive contraction of the diaphragm and the abdominal muscles, which compel it involuntarily to discharge its contents, together with those of the bronchial passages. As a proof that this process takes place without previous sickness, or gastric derangement, the patient, immediately after the fit of coughing is over, feels hungry, and calls for food. In all cases, the approach of the paroxysms excites in the patient a marked apprehension of impending distress, and instinctively propels him to secure himself from falling by seizing hold of a table, or some other firm support, or by attaching himself to his nurse's dress. At first, little or no expectoration occurs, but as the second stage advances, either viscid mucus, or pus is expelled, and terminates the paroxysm. The period at which the purulent secretion commences, is three weeks after the first appearance of the disease, at which time a quotidian or regular evening paroxysm of fever, of the nature of evening hectic fever, symptomatic of the purulent secretion, is discovered. When the patient is carelessly exposed to a cold atmosphere, bronchitis, pneumonia, or pleuro-pneumonia, is superadded to the disease, and protracts its duration; and these complications are associated with cerebral convulsions in plethoric children, who have large heads. When the inflammation attacks the bronchial ramifications, and much mucous secretion follows, they are liable to become dilated, and thus to increase the misery of the patient, both during the disease and after its termination. During pneumonia, also, vesicular or interlobular emphysema may arise, and add to his distress; the former leaving permanent dyspnœa, and the latter endangering infiltration of air through the mediastinum into the cellular membrane of the face, neck, or chest. The violence of the cough may also produce rupture of the capillary vessels of the eyelids and upper lip, greatly disfiguring the patient. In the decline of hooping-cough, the latent disordered state of the alimentary canal, discovers itself in the form of remittent fever in delicate infants, accompanied with emaciation, and sometimes with spasm of the glottis; and this secondary fever aggravates and prolongs the duration of the original disease. During the catarrhal period of hooping-cough in infants and young children, the mucous membrane of the bowels is almost always affected concurrently with slight dysentery, which in the former is erroneously attributed to dentition, and in the latter is overlooked, in consequence of the cough attracting predominant exclusive attention. For a more full explanation of my views, respecting the nature and origin of remittent fever and spasm of the glottis, the reader is referred to the chapters on those diseases. It must, however, be observed, that when the latter disease is connected with epilepsy, there will be reason to suspect the consecutive attack of some cerebral, or, more probably, cerebellous congestion, or inflammation. In scrofulous children, the second stage of hooping-cough is sometimes accompanied with an intense heat and dryness of the skin, alternating with chilliness, which ultimately terminates in hectic fever, and the development of tubercular disease in the lungs.

"It is needless for me to enumerate all the various theories which have been invented to explain the origin and nature of hooping-cough. It appears to me to be nothing more than a bronchial catarrh, of a specific character, which is modified by the treatment and the constitution of the patient; and my observation of, and extensive experience in, the treatment of the disease, induce me to concur with Billard, and other French writers, in considering it to be an epidemic and not a contageous disease. As I before remarked, the hooping-cough begins in some children with inflammation of the larynx. This gradually descends the trachea, until it reaches the bronchi, at the bifurcation of which is seated the most sensitive part of the air-passages. The same process takes place when the disease commences as common catarrh. As soon as the specific inflammation reaches this irritable part, the peculiar running, suffocating cough is observable, and every subsequent exposure to cold increases, extends, and prolongs the disease. It is the opinion of Dr. Copland, that hooping-cough in the simple form, is altogether nervous, and that in uncomplicated cases, the nervous affection never proceeds beyond irritation.* Dr. Webster also believes, that the symptoms depend upon inflammatory irritation in the brain or of its membranes;† and Leroy,‡ Boisseau, Otto,|| and Begin,§ have also observed the frequent connection of cerebral disease with hooping-cough from the beginning of the attack, but they by no means admit that the latter is dependant on the cerebral affection. If the cough were only nervous, we should expect to see it terminate, as the hysterical and other nervous coughs, without expectoration, and without the peculiar, shrill inspiration. In hooping-cough, however simple, we invariably find the patient expectorate either viscid mucus or pus, and we know that in simple, chronic laryngeal inflammation, the cough is followed by the discharge of a starch-like inspissated mucus. The post-mortem appearances consist of increased vascularity, or actual inflammation of the mucous membrane of the air-passages, extending even to the pulmonary air-cells.

"'On examination after death, the usual morbid appearance is inflammation of the mucous membrane. The lungs collapse imperfectly, and when cut into, an abundance of frothy and puriform mucous exudes from the bronchi and air-cells. Increased solidity of the lung has often been found, and by some it is said to be constantly observable. When it does occur, it would appear that the inflammation had extended from the mucous membrane to the substance of the lung, or attacked both its textures.'¶ These morbid appearances are accounted for by Dr. Copland in the following manner:—

"'The impression made by the causes is followed by the lesion of the respiratory nerves, particularly the nervus vagus; and, owing to this lesion, the mucous surfaces they supply, frequently experience consecutive changes, as respects the circulation, exhalation, and secretion. Hence result vascular determination and augmented secretion, attended by irritation of the glottis, epiglottis, pharynx and air-tubes, inducing convulsive action, which supervenes the more readily, as the disease is not only essentially nervous in its nature, but often becoming consecutively irritative, or inflammatory; this last characteristic being only an occasional complication, occurring from predisposition, habit of body, epidemic influence or fortuitous causes favourable to its development.'**

"In opposition to this theory it may be observed, first, that

* "Dict. of Pract. Med." part v., p. 242. † "Med. and Phys. Journal," Dec. 1822.

‡ "Med. Maternelle," Paris, 1803. || "Nye Hygæa," Aug., 1824.

§ "Traité de Therapeutique," &c., tom. ii., 1825.

¶ Dr. C. Johnson, "Cyclop. of Practical Med.," vol ii., p. 430.

** Loco citato, p. 243.

when the cough is slight, as in most adults, no concomttant cerebral symptom is present; secondly, that after an attack of spasm of the glottis, which is now acknowledged is produced by the excited action of the pneumogastric nerve, we can neither discover any expectoration during life, nor laryngeal, tracheal, or bronchial inflammation after death; thirdly, that the disease always commences with inflammation in the bronchial or some other portion in the mucous membrane lining the air passages, as other epidemic catarrhs; and, fourthly, that the symptoms of cerebral or cerebellous disease never unfold themselves until the second stage, denoted by the spasmodic cough, has established itself. Hence it appears to me, that the brain and cerebellum are affected in a secondary manner by the temporary obstruction in the pulmonary circulation occurring during the paroxysms of convulsive cough, and the impediment to the return of the venous blood from the brain and consequent cerebral congestion. Adopting this view of the pathology of hooping-cough, it will be found that its treatment may be facilitated, its duration limited, and its severity and danger greatly diminished by the practice which I have long adopted, and am about to recommend.

"*Treatment.*—As soon as the disease is discovered to be hooping-cough, the patient must be confined day and night to a temperature of sixty-five degrees of Farhenheit's thermometer. This degree of temperature may be artificially raised and maintained in most houses. The temperature must be the same in the bed-room as in the sitting-room, and both rooms should, if possible, be on the same floor. The bed-room should be ventilated during the day, and the sitting-room during the night; but the windows of the apartments must on no account be opened while the patient is in them. The bowels may be regulated by some gentle aperient, as salts and senna. No other medicine will be required during the first stage of the disease, except a mixture composed of citrate of potash and squill. When the second stage arrives, while proper attention is paid to temperature, the cough will be found much slighter, and the expectoration much less than if the child were permitted to be exposed to the external air; and at the end of six or eight weeks at the farthest, all symptoms of the disease will disappear. This regulated temperature may be commenced at any stage of the disease with advantage, while the cough is alarming, and the expectoration copious or purulent; and it will not interfere with any treatment which bronchial or pulmonary inflammation may specially demand. Should the disease have been neglected, and the patient be found suffering with purulent expectoration and hectic fever, before the regulation of the temperature has been adopted, he may be speedily relieved by adhering to it, and by the exhibition of half a grain or a grain of sulphate of zinc, or a quarter or a half a grain of sulphate of copper, dissolved in an ounce of water, with half a grain of disulphate of quina, three times a-day. These metallic sulphates have, with the assistance of an exalted temperature, the effect of reducing the mucous and purulent secretion in this disease, on the same principle on which they succeed in the cure of chronic nasal catarrh, to which the reader is referred. The quina will assist the stomach in retaining the zinc or copper, and in removing the periodicity or quotidian access of the fever. Acute bronchial and pulmonary inflammation must be treated by suitable venesection and other means adapted to these diseases; but they will never be found to arise, when the regulation of temperature is uninterruptedly employed from the commencement of the disease.* I have before stated, that the mucous membrane of the bowels is frequently affected simultaneously with that of the bronchi or larynx. Hence we must expect remittent fever to manifest itself towards the decline of the bronchial disease. The earliest symptoms of this consecutive fever, will be moaning in the sleep, rapid emaciation, picking of the lips and fingers, and rubbing of the eyes, with which will be associated peevishness, perverseness, and disinclination for amusements. In this state, the least annoyance or opposition excites passion, which is immediately followed by a fit of coughing. The treatment of this modification as well as that of spasm of the glottis, when that is also complicated with hooping-cough, must be conducted in the same manner as I have directed, when speaking of these complaints. Patients who may have had their illness prolonged by either of these diseases should be removed to a healthy situation, where they may enjoy a pure, mild air, as soon as the disordered state of the bowels has been removed. Should any cough remain, which is sometimes kept up by habit and local association, the change of air and scene will at this period soon remove it. The most common convulsions excited by the violent paroxysm of hooping-cough are those which constitute epilepsy. As these almost invariably appear only in robust children, leeches must be applied to the temples, and the bowels freely opened; and if the epilepsy should still persist, the warm bath may be prescribed. If the child is of sufficient age to admit of venesection, he may be bled at the arm; if not, and the case is urgent, the jugular vein should be opened, and three or four ounces of blood abstracted. These are the most dangerous and fatal

* After the removal of the complication of pneumonia or of bronchitis and bronchial congestion, and if the hoop be long and frequent, and still more, if there be tendency to glottic spasm, or to convulsions, assafœtida and balladonna, the first in the form of mixture, the latter in that of tincture, will be found to be valuable remedies. To each are added advantageously, the carbonate of potassa and ipecacuanha wine. Frictions with stimulating liniments along each side of the spinal ridge are a popular and a useful addition to the general treatment.

convulsions, to which children are liable, and therefore relief must be promptly afforded. Hydrocephalus rarely succeeds an attack of hooping-cough. When it does occur, it must be treated by the usual remedies for that disease. In scrofulous children the bronchial inflammation sometimes terminates in the development of tubercles, which hurry the patient into pulmonary consumption. On this account, the progress of hooping-cough should be carefully watched, in order that acute inflammation in the mucous membrane, and in the pulmonary parenchyma, may be discovered and immediately relieved. Should latent phthisis be detected in the decline of the disease, the patient should be removed to a warm climate, which, in many cases, will have the effect of suspending or retarding the progress of tuberculisation."

The treatment recommended in the above extract, has been tried with favorable results, by a considerable number of persons, and if the reports given of its efficiency, should be sustained, it will certainly form a most useful discovery. The same may be said, too, of the strychnine used in incontinence of urine.

"*Incontinence of Urine, or Involuntary Discharge of Urine.*—An involuntary flow of urine during sleep, is one of the most disagreeable and unfortunate infirmities to which children are liable. It is occasioned by a partial paralysis of the sphincter muscle of the bladder, which derives its nervous influence from the medulla spinalis. The attack usually occurs during the first sleep, when volition is dormant, and the excitomotory system uncontrolled by any counteracting influence. In most patients, a derangement in the functions of the stomach and bowels, which produces general muscular atony and diminished vital energy, from the imperfection of the primary process of sanguification, may be detected by loss of appetite and animal spirits, paleness of the countenance, indisposition for exercise or amusement, furred tongue, and an offensive and unnatural state of the instestinal discharges. To these symptoms may be added itching of the nostrils, lips, and eyelids, and irritability of temper.

"Incontinence of urine also occurs in some children who are subject to epileptic attacks during the night. In these cases it is probable that paralysis of the bladder is occasioned by temporary congestion in the cerebellum or medulla oblongata, promoted by sleep and the recumbent position.

"*Treatment.*—This is general and special. The general treatment consists in the restoration of the chylopoietic derangement, by proper purgatives, as chloride of mercury and jalap administered every third morning; or a few grains of the former medicine every third night, and a dose of salts and senna the following morning. The epileptic or plethoric should be limited to proper diet, and all descriptions of patients should be restricted from food, and especially from liquids, during several hours before bed-time.

"The best special treatment will be found in the administration of strychnine or nux vomica, which medicines exercise a specific power over the spinal nerves, which they excite into action in a most extraordinary manner. The dose of strychnine for a child, from five to ten years of age, is one-twelfth of a grain, and that of finely powdered nux vomica two grains, three times a-day. The curative effect of either of these medicines is so infallible that I never have occasion to prescribe any other specific remedy."

We shall here close our notices of this work which has consisted principally in extracting such passages as might serve at once to instruct our readers and show the merits of the book. It is the most full and perfect treatise with which we are acquainted on the surgical diseases of infants, and not deficient in what relates to the medical department of infantile pathology.

